# Supplementary material for: Drug repurposing for aging research using model organisms
Source: Aging Cell. 2017 Jun 16;16(5):1006–15. doi: 10.1111/acel.12626 (PMC5595691; doi:10.1111/acel.12626)
Supplement: Supplementary file 7 — Data S1 Zip‐Archive of all report cards. [file ACEL-16-1006-s007.zip › RC_QCT.pdf]

QCT

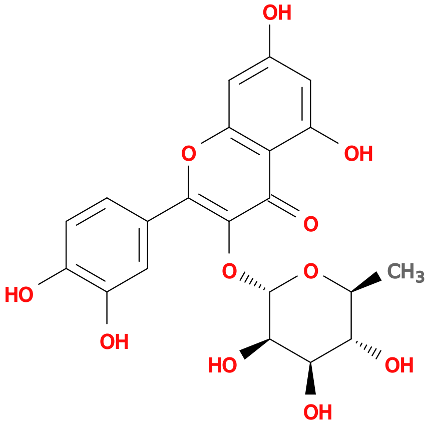

Database identifiers

ChEMBLCompound	CHEMBL82242
CHEBI	17558
ZINC	ZINC04175638
eMolecules	531297

Ranking

	Rank	Score
Drosophila	236/697	0.666
C. elegans	307/591	0.189

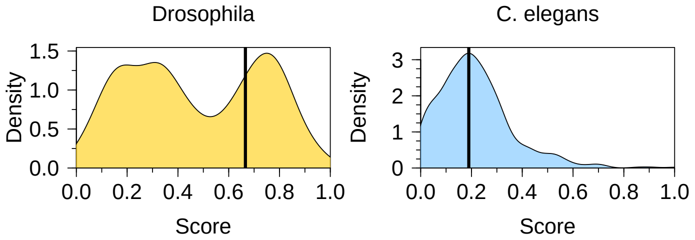

	Ageing implication	Domain conservation	Binding site conservation	Binding affinity	Bioavailability	Lipinski	Promiscuity	Purchasability	Drug approval	**Total**
Drosophila	1.0	0.964	1.0	0.768	(0.9)	-0.1	-0.0	0.1	0.0	0.666
C. elegans	1.0	0.968	1.0	0.768	0.255	-0.1	-0.0	0.1	0.0	0.189

Names

- **quercitrin**
- 3,3',4',5,7-Pentahydroxyflavone-3-L-rhamnoside
- luteolin 6-deoxy-alpha-L-mannopyranoside
- Quercetin 3-O-alpha-L-rhamnopyranoside
- Quercetin 3-O-rhamnoside
- Quercimelin
- Quercitronic acid
- Quercitroside

Roles

antioxidant, antileishmanial agent, metabolite

Status

Approved drug (according to ChEMBL)	No
Number of Rule of 5 violations	2
Binding affinity to original target in log units (RF-Score prediction)	6.2
Burns *C. elegans* bioavailability prediction	-1.84

Compound Target Characteristics

Ribosomal protein S6 kinase alpha-3

Best gene implication in ageing for this target family came from gene Q86NP0 annotated in UniProt release 2014_02. Annotation GO 8340 (determination of adult lifespan) was Inferred from Mutant Phenotype

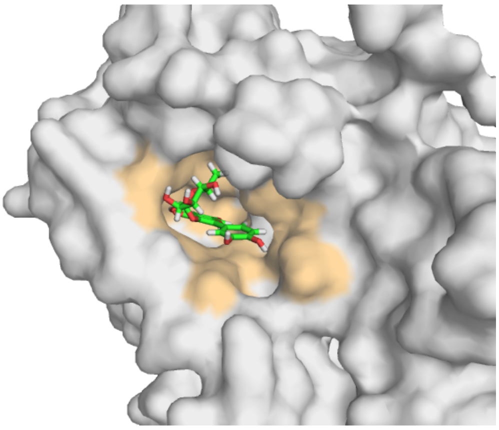

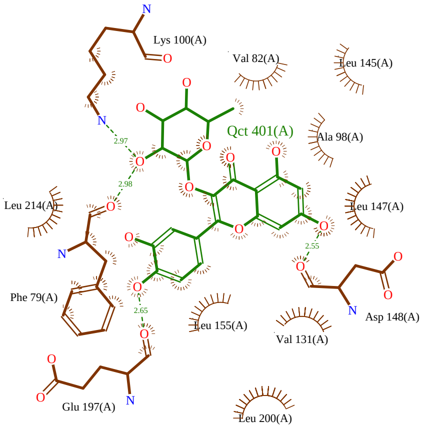

protein	amino acids contacts (binding site)
PDB:4gue:chainA:P18654	F V A K V L L D L E L L
tr:B1AXG1:B1AXG1_HUMAN	F V A K V L L D L E L L
tr:B7Z2K7:B7Z2K7_HUMAN	F V A K V L L D L E L L
tr:B4DG22:B4DG22_HUMAN	F V A K V L L D L E L L
tr:B7ZB17:B7ZB17_HUMAN	F V A K V L L D L E L L
tr:E9PRI4:E9PRI4_HUMAN	F V A K V L L D L E L L
tr:F5GYC4:F5GYC4_HUMAN	F V A K V L L D L E L L
tr:E9PGT3:E9PGT3_HUMAN	F V A K V L L D L E L L
sp:Q15418:KS6A1_HUMAN	F V A K V L L D L E L L
sp:P51812:KS6A3_HUMAN	F V A K V L L D L E L L
tr:F1LXV0:F1LXV0_RAT	F V A K V L L D L E L L
tr:D3Z8E0:D3Z8E0_RAT	F V A K V L L D L E L L
tr:B1AXP0:B1AXP0_MOUSE	F V A K V L L D L E L L
tr:B1AXN9:B1AXN9_MOUSE	F V A K V L L D L E L L
tr:E9PWV3:E9PWV3_MOUSE	F V A K V L L D L E L L
sp:P18653:KS6A1_MOUSE	F V A K V L L D L E L L
tr:F6RQA2:F6RQA2_MOUSE	F V A K V L L D L E L L
tr:Q505N6:Q505N6_MOUSE	F V A K V L L D L E L L
tr:F6Q8A4:F6Q8A4_MOUSE	F V A K V L L D L E L L
sp:P18654:KS6A3_MOUSE	F V A K V L L D L E L L
tr:Q810V8:Q810V8_MOUSE	F V A K V L L D L E L L
tr:Q86NP0:Q86NP0_DROME	F V A K V L L D L E L L
tr:Q9VR61:Q9VR61_DROME	F V A K V L L D L E L L
tr:Q24496:Q24496_DROME	F V A K V L L D L E L L
sp:Q21734:KS6A1_CAEEL	F V A K V L L D L E L L
tr:G5EE78:G5EE78_CAEEL	F V A K V L L D L E L L

protein	whole protein ident	simil	domain-based ident	simil	contact-based ident	simil
PDB:4gue:chainA:P18654	1.0	1.0	1.0	1.0	1.0	1.0
tr:B1AXG1:B1AXG1_HUMAN	0.28	0.29	0.72	0.72	1.0	1.0
tr:B7Z2K7:B7Z2K7_HUMAN	0.65	0.73	0.9	0.97	1.0	1.0
tr:B4DG22:B4DG22_HUMAN	0.96	0.96	1.0	1.0	1.0	1.0
tr:B7ZB17:B7ZB17_HUMAN	0.96	0.96	1.0	1.0	1.0	1.0
tr:E9PRI4:E9PRI4_HUMAN	0.76	0.88	0.9	0.97	1.0	1.0
tr:F5GYC4:F5GYC4_HUMAN	0.94	0.95	1.0	1.0	1.0	1.0
tr:E9PGT3:E9PGT3_HUMAN	0.73	0.84	0.86	0.93	1.0	1.0
sp:Q15418:KS6A1_HUMAN	0.74	0.85	0.9	0.97	1.0	1.0
sp:P51812:KS6A3_HUMAN	1.0	1.0	1.0	1.0	1.0	1.0
tr:F1LXV0:F1LXV0_RAT	0.74	0.85	0.9	0.97	1.0	1.0
tr:D3Z8E0:D3Z8E0_RAT	1.0	1.0	1.0	1.0	1.0	1.0
tr:B1AXP0:B1AXP0_MOUSE	0.27	0.27	0.66	0.66	1.0	1.0
tr:B1AXN9:B1AXN9_MOUSE	0.96	0.96	1.0	1.0	1.0	1.0
tr:E9PWV3:E9PWV3_MOUSE	0.76	0.87	0.91	0.97	1.0	1.0
sp:P18653:KS6A1_MOUSE	0.73	0.84	0.87	0.93	1.0	1.0
tr:F6RQA2:F6RQA2_MOUSE	0.75	0.85	0.91	0.97	1.0	1.0
tr:Q505N6:Q505N6_MOUSE	0.75	0.85	0.91	0.97	1.0	1.0
tr:F6Q8A4:F6Q8A4_MOUSE	0.74	0.85	0.91	0.97	1.0	1.0
sp:P18654:KS6A3_MOUSE	1.0	1.0	1.0	1.0	1.0	1.0
tr:Q810V8:Q810V8_MOUSE	0.71	0.81	0.91	0.97	1.0	1.0
tr:Q86NP0:Q86NP0_DROME	0.46	0.68	0.78	0.93	1.0	1.0
tr:Q9VR61:Q9VR61_DROME	0.46	0.69	0.78	0.93	1.0	1.0
tr:Q24496:Q24496_DROME	0.47	0.69	0.78	0.93	1.0	1.0
sp:Q21734:KS6A1_CAEEL	0.55	0.79	0.81	0.94	1.0	1.0
tr:G5EE78:G5EE78_CAEEL	0.53	0.77	0.81	0.94	1.0	1.0

S6kII (FBgn0262866) associated phenotypes

dominant, learning defective, male limited, memory defective, neuroanatomy defective, reduced, short lived

(Information from FlyBase)

rskn-1 (UniProt:Q21734) annotation

Function: Serine/threonine kinase that may play a role in mediating the growth-factor and stress induced activation of transcription.
Cofactor:Mg(2+)
Enzyme regulation: Activated by multiple phosphorylations on threonine and serine residues.
Ptm: Autophosphorylated on Ser-422, as part of the activation process.

(Information from UniProt)

rskn-1 (UniProt:G5EE78) annotation

Cofactor: Mg(2+) Evidence=(PIRNR:PIRNR000606);

(Information from UniProt)
